# Screening of the Dichloromethane: Methanolic Extract of *Centella asiatica* for Antibacterial Activities against *Salmonella typhi*, *Escherichia coli*, *Shigella sonnei*, *Bacillus subtilis*, and *Staphylococcus aureus*

**DOI:** 10.1155/2020/6378712

**Published:** 2020-07-01

**Authors:** Berick Moturi Sieberi, George Isanda Omwenga, Rachael Kitondo Wambua, Judith Chemutai Samoei, Mathew Piero Ngugi

**Affiliations:** Department of Biochemistry, Microbiology and Biotechnology, School of Pure and Applied Sciences, Kenyatta University, 43844-00100 Nairobi, Kenya

## Abstract

Bacterial infections are responsible for a large number of deaths every year worldwide. On average, 80% of the African population cannot afford conventional drugs. Moreover, many synthetic antibiotics are associated with side effects and progressive increase in antimicrobial resistance. Currently, there is growing interest in discovering new antibacterial agents from ethnomedicinal plants. About 60% of the population living in developing countries depends on herbal drugs for healthcare needs. This study involved the screening of *Centella asiatica* commonly used by herbal medicine practitioners in Kisii County to treat symptoms related to bacterial infections. Standard bioassay methods were applied throughout the study. They included preliminary screening of dichloromethane: methanolic extract of *Centella asiatica* against human pathogenic bacteria including *Salmonella typhi* ATCC 19430, *Escherichia coli* ATCC 25922, *Shigella sonnei* ATCC 25931, *Bacillus subtilis* ATCC 21332, and *Staphylococcus aureus* ATCC 25923 using agar disc diffusion, broth microdilution method, and time-kill kinetics with tetracycline as a positive control. Phytochemical screening was carried out to determine the different classes of compounds in the crude extracts. Data were analyzed using one way ANOVA and means separated by Tukey's test. Dichloromethane: methanolic extract of *Centella asiatica* was screened against the selected bacterial strains. Time-kill kinetic studies of the extracts showed dose- and time-dependent kinetics of antibacterial properties. Phytochemical screening of the DCM-MeOH extract revealed the presence of alkaloids, flavonoids, phenolics, terpenoids, cardiac glycosides, saponins, steroids, and tannins. The present study indicates that the tested plant can be an important source of antibacterial agents and recommends that the active phytoconstituents be isolated, identified, and screened individually for activities and also subjected further for *in vivo* and toxicological studies.

## 1. Introduction

Infectious diseases are a major cause of morbidity and mortality worldwide [1–3]. The use of antibiotics to control these diseases has led to the emergence of antibiotic-resistant pathogens and, therefore, the need for alternative medicines [4, 5], such as medicinal plants [6], which are easily available, affordable, and efficacious with minimal side effects [7, 8]. The problem of antibiotic resistance is confounded by the emergence of “superbugs” such as *Staphylococcus aureus*, *Mycobacterium tuberculosis*, *Enterobacter* species, *Klebsiella pneumoniae*, *Acinetobacter baumannii*, and *Pseudomonas aeruginosa* that are resistant to multiple classes of antibiotics [9, 10].

In addition, many bacterial pathogens form biofilms when they come in contact with a hydrated surface [11]. The biofilms are extracellular matrices that enclose aggregates of bacterial cells on surfaces [11, 12] and are a major problem in clinical therapeutics since microbial communities adhered to surfaces are physiologically distinct from planktonic cells of the same bacteria [13]. The formation of bacterial biofilms has been shown to increase resistance to antibiotics by up to 1000-fold [14]. In bacterial culture, a growth medium is required which is a culture media either in a solid, liquid, or semisolid form designed to support the growth of bacteria. A culture media used for general cultivation and maintenance of bacteria contains a carbon source, water, salts, amino acids, and nitrogen [15].

About 80% of the population living in developing countries uses medicinal plants for their health care needs due to their inability to maintain a steady supply of conventional medicines [7, 16]. In some cases, herbal drugs are used in combination with conventional drugs if the patient feels that the prescribed medicines are ineffective [17].

The World Health Organization (WHO) emphasizes the need to compliment conventional treatment with herbal medicines [18], and through its sensitization and mobilization programs, African countries have been encouraged to begin serious advancements in herbal medicine use in order to sustain provision of healthcare and ensure continuity of culture [16]. Exploiting plant extracts with known antibacterial activities is significant in managing various infectious diseases [19].

Bacterial resistance to currently used antibiotics necessitates the search for effective therapeutic agents. The use of medicinal plants presents a great potential as a source of antimicrobial compounds against resistant pathogenic microorganisms [20].

Medicinal plants have been used for a long time [21] and have a track record of being effective, safe, and cheap to use [22]. For instance, *Glycyrrhiza glabra* has been used for the management of respiratory ailments such as coughs, sore throat, and bronchitis [23] and *Mahonia aquifolium* has been used in skin infection management [24], while *Achillea millefolium* and *Arctostaphylos uva-ursi* are used to manage urinary tract infections [25, 26].

A large number of people in developing countries depend on medicinal plants as their primary source of medication [27].

*Centella asiatica* has been utilized traditionally for the treatment of infectious diseases among the Abagusii community in Kenya, with limited scientific documentation of its dichloromethane: methanolic extract antibacterial activities. This study aimed at screening the dichloromethane: methanolic extract of *Centella asiatica* for antibacterial activities against selected bacterial pathogens.

*Centella asiatica* is used to treat wounds, mental and neurological disorders, atherosclerosis, microbial infections, and cancer [28]. It is also used in the treatment of inflammations, diarrhea, asthma, tuberculosis, and various skin lesions and ailments such as leprosy, lupus, psoriasis, and keloid. It is reported to possess ulcer-preventive, antioxidant, and antidepressive effects and improves venous insufficiency. An alcoholic extract of the whole plant showed antiprotozoal activity against *E*. *histolytica* [28], while a chloroform extract of the whole plant showed activity against *Bacillus subtilis, Staphylococcus aureus, Bacillus cereus, Escherichia coli, Salmonella typhi,* and *Shigella dysenteriae*.

**Figure 1:**
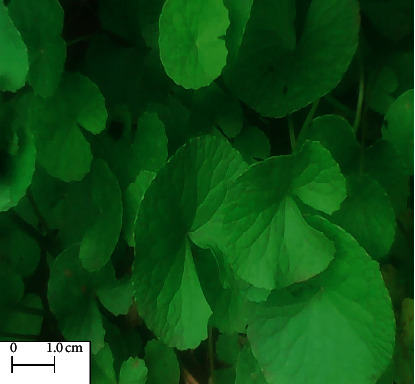
Picture of *Centella asiatica* L.

*Centella asiatica* L. is a member of the family Umbeliferae; the photograph of the plant is presented in Figure 1. It is a perennial herbaceous creeper that grows in moist areas and is distributed widely in tropical and subtropical countries [29]. It has a faint aroma with white to pink flowers and 1–3 leaves of sheathing base from each node. The leaves are smooth with parallel lines on the surface and roots at the stem nodes. The fruits are about 2 inches long and spherical shaped with a thick pericarp. Its seeds consist of a pedulous embryo that looks compressed [29–31]. Vernacular names include English- Indian pennywort, Hindi- Gotukolu, and Chinese- Fo-ti-tieng, while in Kenya, it is referred to as Mungutab beliot ne sing'ortot (nandi) and Enyonyo engare (Kisii).

## 2. Materials and Methods

### 2.1. Plant Sample Collection and Preparation

Whole plant samples of *Centella asiatica* were sourced from Masaba south sub-County in Kisii County, Kenya, under accepted bioconservation methods with the help of a local herbalist. A fresh sample was botanically authenticated by an acknowledged taxonomist, Mr. Lucas Karimi, from the Pharmacy Department, Kenyatta University, and assigned a voucher specimen number BM/26345/001/2016. A sample voucher was deposited at the Department of Pharmacy research herbarium of Kenyatta University. The plant samples were transported to Kenyatta University, Department of Biochemistry, Microbiology and Biotechnology laboratories, washed with clean water to remove soil, and dried under shade for 14 days. The samples were then milled into a fine powder with an electric mill. The powder was weighed and stored at room temperature in airtight containers, awaiting extraction.

### 2.2. Extraction

Two hundred grams (200 g) of the plant sample powder was weighed and soaked in a cold 600 ml mixture of dichloromethane: methanol (1 : 1) for 48 h in a flask to obtain the extract [32]. After extraction, it was decanted and, then, filtered using Whatman No. 1 filter papers, and the filtrate was concentrated under vacuum using a rotary evaporator at 40°C. The extract obtained was kept in an airtight container and stored at −20°C for later use in an antimicrobial assay.

### 2.3. Experimental Design

A completely randomized design was applied in this study.

### 2.4. Tested Microorganisms

Microorganisms used in the experiment were standard reference strains sourced from the Microbiology laboratory, Kenyatta University, namely, *Salmonella typhi* (ATCC 19430), *Escherichia coli* (ATCC 25922), *Shigella sonnei* (ATCC 25931), *Bacillus subtilis* (ATCC 21332), and *Staphylococcus aureus* (ATCC 25923).

#### 2.4.1. Maintenance of Microbial Stock Cultures

The stock cultures of bacterial strains were cultured on Mueller Hinton agar and incubated at 37°C for 24 h to obtain fresh growing colonies [33]. Three to four colonies were transferred using a sterile wire loop into sterile capped glass tubes with 10 ml of sterile Mueller Hinton broth and incubated at 37°C for 24 h to obtain a fresh growing bacterial suspension and maintained at 4°C.

### 2.5. Preparation of Plant Extract Dilutions and Impregnated Antimicrobial Agent Discs

The plant extract stock solution was reconstituted using 0.2% DMSO by weighing 1 g of the extract in a sterile sample bottle and dissolved in 1 ml 0.2% DMSO to make a concentration of 1 g/ml. The stock solution was then diluted serially in a two-fold dilution starting from 500 mg/ml. Paper discs of 6 mm in diameter were prepared from Whatman No. 1 filter paper using a paper punch and sterilized at 121°C for 15 min. Twenty microlitres from each of the different concentrations was used to impregnate the paper discs. The impregnated discs were air dried in a laminar flow hood for 3 h and immediately used for the sensitivity tests.

### 2.6. Antimicrobial Sensitivity Test Using the Disc Diffusion Method

The assays for antimicrobial activity of the DCM: methanolic extract of *C. asiatica* were performed by the disc diffusion method [34]. Bacteria from the stock cultures were cultured overnight at 37°C in MHA and used as the inoculum. Bacterial cultures were adjusted to 0.5 McFarland's standard to achieve a concentration of 1.5 × 10^8^ CFU/ml in physiological saline and then used to lawn Mueller–Hinton agar plates evenly using sterile cotton swabs. The inoculated plates were air-dried in the laminar airflow hood for 15 min and, then, used for the sensitivity test. The discs, impregnated with a series of plant extract dilutions, were placed on the Mueller–Hinton agar surface with each test plate comprising eight discs equidistant from each other. A standard commercial antibiotic disc (Tetracycline 30 *µ*g) was used as a positive control, while a disc impregnated with 0.2% DMSO was used as the negative control. The plates were incubated at 37°C for 24 h after which zones of inhibition around the discs were observed and measured. The tests were performed in triplicates.

### 2.7. Determination of Minimum Inhibitory Concentrations (MICs)

The broth microdilution method was used to determine minimum inhibitory concentrations (MICs) of the plant extract against the test bacterial cultures using 96-well microtiter plates [35]. Starting from the highest concentration of each extract, two-fold serial dilutions were prepared in sterile MHB in sterile test tubes, resulting in a concentration range from 500 mg/ml to 7.8125 mg/ml, the wells were clearly labeled with the respective extract concentration, and 50 *µ*l of each extract dilution was added into the respective well for each bacterial isolate to be tested. Each well containing the extract dilution and the growth control well was inoculated with 50 *µ*l of the bacterial suspension to achieve a final inoculum of 5 × 10^5^ CFU/ml, and the plate was incubated at 37°C for 24 h.

Ten microlitre (10 *μ*l) samples from the growth control well were removed immediately after inoculation and mixed with 990 *μ*l of sterile MHB in a sterile microcentrifuge tube. This suspension was further diluted by taking 100 *µ*l and adding to 900 *µ*l sterile MHB. A hundred microlitres (100 *µ*l) from each of the dilutions was plated on nutrient agar and incubated at 37°C for 24 h together with the microtiter plate. Colonies were counted after 24 h of incubation to confirm the CFU/ml present in the innocula.

The MIC was recorded as the lowest concentration of the extract that inhibited visible growth of the test bacteria as observed by an unaided eye. Each experiment was performed in triplicate.

### 2.8. Determination of Minimum Bactericidal Concentrations (MBCs)

Samples of 10 *μ*l from each well with a concentration at and above the MIC of the antimicrobial agent being tested [32] were taken, and the inoculum was spread over the MHA plate with a sterile cotton swab [36]. The plates were incubated at 37°C for 24 h. After incubation, visible colonies were counted on each subculture plate, as well as the initial inoculum plates. The MBC was recorded as the lowest concentration of the plant extract in which there was a reduction of the initial inoculum by 99.9% [37].

### 2.9. Determination of Time-Kill Kinetics

Time-kill kinetics was carried out to indicate the rate and extent of bacterial killing by the antibacterial agent [38], based on the MIC and MBC reactions [34].

#### 2.9.1. Preparation of the Inoculum

Twenty-four hour cultures of the test microorganisms on MHA were suspended in MHB to obtain bacterial suspension of 1 × 10^6^ CFU/ml. One microlitre (1 ml) of the bacterial suspension was added to 9 ml of MHB containing the extract.

#### 2.9.2. Plant Extract Preparation

The *C. asiatica* DCM: methanolic extract was prepared in MHB to a final concentration corresponding to 1 × MBC, 2 × MBC, 3 × MBC, and 4 × MBC. From each concentration, 9 ml was mixed with 1 ml of the inoculum prepared as mentioned above and incubated at 37°C. A growth control with only the bacterial cultures in MHB and a culture with tetracycline drug were used as a negative and positive control, respectively. Five hundred microlitre samples were taken from each test tube at times of 0, 0.5, 1, 2, 3, 4, 6, 8, 12, and 24 h after incubation and diluted tenfold in physiological saline. A hundred microlitres (100 *µ*l) of each dilution was plated on MHA and plates incubated at 37°C for 24 h. Growing colonies were counted after 24 h of incubation with only plates containing 30–300 colonies of each dilution counted. Growth curves were plotted to show the log_10_ of colony forming units (CFU) against time [39, 40].

### 2.10. Qualitative Phytochemical Screening

Qualitative phytochemical screening was performed on the plant extract to determine the presence or absence of bioactive compounds [41].

### 2.11. Statistical Analysis

Experimental data on zones of inhibition readings, MIC, MBC, and time-kill kinetics were recorded and tabulated on a broad spread sheet using MS excel program. Statistical analysis of the data was performed using Minitab statistical software version 17.0.

In order to test for parametric assumptions, results were expressed as mean ± standard error of the mean (SEM). One-way ANOVA and Tukey's post-hoc test were used for separation and comparison of means to obtain the specific significance difference. The values of *p* ≤ 0.05 were considered to be significant. The time-kill kinetics plot of log_10_ CFU against time was drawn to determine the time required to kill all the bacteria.

## 3. Results

### 3.1. Antibacterial Sensitivity of the Extract

In this study, the dichloromethane: methanolic extracts of *C. asiatica* demonstrated antibacterial activities against *E. coli*, *S. typhi*, *S. aureus*, *B. subtilis*, and *S. sonnei.* Tetracycline (30 *µ*g) was used as a positive control, and 0.2% DMSO was used as a negative control. The antibacterial effects of the reference drug (tetracycline) were significantly higher against the five bacterial species compared with the *C. asiatica* extract at all concentrations tested (*p* < 0.05; Table 1). The inhibitory activities of the *C. asiatica* extract concentration of 500 mg/ml were significantly higher against the five bacterial species tested compared to the other extract concentrations tested (*p* < 0.05; Table 1). The same trend was observed when *C. asiatica* extracts were tested at 250 mg/ml and 125 mg/ml. At the lower *C. asiatica* extract concentrations, there were no significant inhibitory activities that were recorded against the test bacterial species; thus, the activity was dose-dependent (*p* < 0.05; Table 1).

### 3.2. Minimum Inhibitory Concentrations (MICs) and Minimum Bactericidal Concentrations (MBCs)

The MIC values obtained for the DCM : MeOH extract of *C. asiatica* against *Salmonella typhi* and *Escherichia coli* were significantly higher compared to those against *S. aureus* (*p* < 0.05; Table 2). The MIC values of the extract against *S. sonnei, B. subtilis*, and *S. aureus* were not significantly different from each other (*p* < 0.05; Table 2). The MBC values obtained for the extract against *Salmonella typhi* and *E. coli* were not significantly different from each other, but were significantly higher than the MBC obtained for *Staphylococcus aureus, Shigella sonnei*, and *B. subtilis* (*p* < 0.05; Table 2).

### 3.3. Time Kill Kinetics

**Figure 2:**
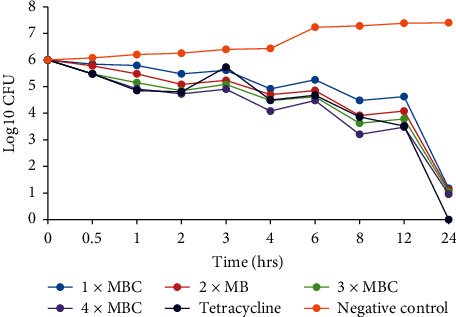
Time-kill kinetics activities of the dichloromethane: methanolic extract of *C. asiatica* against *E. coli*.

**Figure 3:**
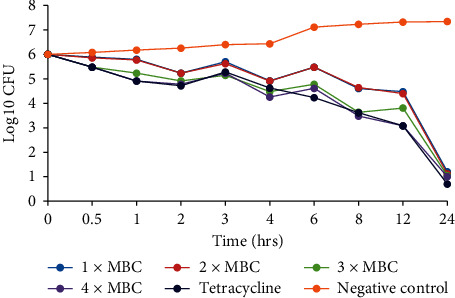
Time-kill kinetics activities of the dichloromethane: methanolic extract of *C. asiatica* against *S. typhi*.

**Figure 4:**
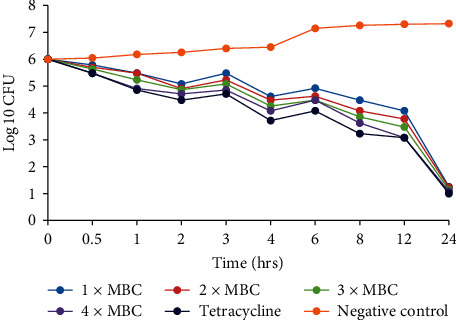
Time-kill kinetics activities of the dichloromethane: methanolic extract of *C. asiatica* against *Shigella sonnei*.

**Figure 5:**
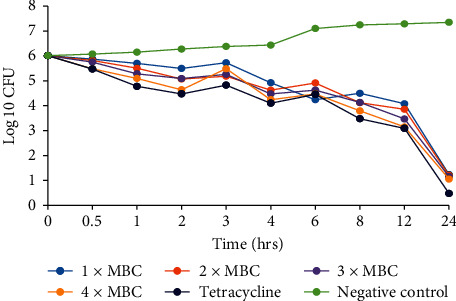
Time-kill kinetics activities of the dichloromethane: methanolic extract of *C. asiatica* against *S. aureus*.

**Figure 6:**
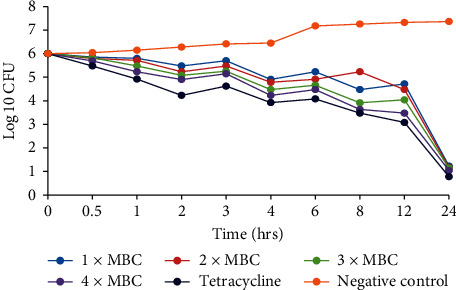
Time-kill kinetics activities of the dichloromethane: methanolic extract of *C. asiatica* against *B. subtilis*.

The time-kill kinetics of the extract concentrations of *C. asiatica* showed similar trends in all the bacterial species tested, with the log_10_ cfu declining with time (Figures 2–6). In all the bacterial species, there were slow log_10_ CFU decline from 0 to 12 h followed by a drastic reduction in log_10_ CFU at the 24^th^ hour.

### 3.4. Phytochemical Screening

Qualitative phytochemical screening of the dichloromethane: methanolic extract of *C. asiatica* revealed the presence of alkaloids, flavonoids, phenolics, tepenoids, cardiac glycosides, saponins, and tannins, but steroids were absent (Table 3).

## 4. Discussion

The inhibition of *S. aureus, E. coli, S. typhi, B. subtilis*, and *Shigella sonnei* by the DCM: MeOH extract of *C. asiatica* shows that this extract has antibacterial compounds. This extract was more potent on Gram-negative than on Gram-positive strains, thus acting as a broad spectrum. These findings are consistent with other reported research works, and this could be attributed to the presence of a lipophilic outer membrane consisting of lipopolysaccharide molecules with an affinity for lipophilic molecules [42]. The phytochemicals present in this extract have been reported to inhibit the growth of both Gram-negative bacteria such as *E.coli, V. cholera, P. aeruginosa, S. senftenberg*, and *Shigella dysenteriae* and Gram-positive such as *S. aureus, S. mutans, S. pneumoniae, B. anthracis*, and *B. subtilis* [8, 43–47]. Thus, they are acting as a broad spectrum similar to tetracycline activities.

The results obtained showed that the extract contained different phytochemicals that included alkaloids, cardiac glycosides, saponins, tannins, flavonoids, terpenoids, and phenols.

Phenolic compounds have been reported to have an antibacterial activity against *Staphylococcus aureus* [8], while flavonoids such as quercetin have been reported to completely inhibit the growth of *S. aureus* [46], and catechins have been reported to have *in vitro* activity against *V. cholerae*, *Streptococcus mutans*, and *Shigella*. Quercetin inhibits *E. coli* as naringenin and sophoraflavanone *G* have been reported to have intensive activity against MRSA and streptococci [45]. Terpenes in cinnamon oil have been observed to inhibit *P. aeruginosa* [47]. Tannin extracts have been reported to inhibit *S. aureus, B. subtilis*, and *Shigella dysenteriae* and, therefore, act as a broad spectrum antimicrobial, while 2, 3, 4-tetrakis-*α*-D-glucosylpyranose is reported to inhibit *S. aureus* [44].

Alkaloids such as ramiflovines A and B extracted from *Aspidosperma ramiflovum* were found to be significantly active against *S. aureus* and *E. faecalis* [47], while cryptolepine and quindoline from *Sida acuta* are reported to be active against *S. aureus, S. dysenteriae, B. cereus. E. faecalis*, and *E. coli*, thus acting as a broad spectrum [48], and Saponin from *Sorghum bicolor* L. Moenh was reported to be active against *S. aureus* [49].

Where similar MIC and MBC values against test bacteria were obtained, MIC indicated a bactericidal activity, while those having MBC greater than MIC, the MIC of the extract, in this case, indicated bacteriostatic activity. From all the tests, the values of MBCs obtained were not more than 4 times higher than those of MICs on the corresponding test bacteria indicating that the extracts tested had an antimicrobial activity [42].

Time-kill kinetics studies of the dichloromethane: methanolic extract of *C. asiatica* at varying concentrations on the test bacteria showed a slow dose- and time-dependent kinetics of killing for both Gram-negative and Gram-positive bacteria. Dose-dependent kinetics occur when the antibacterial agent is at a high concentration at the target site for it to kill the bacteria, while time-dependent kinetics is observed when the concentration of the antibacterial agent is more than the MIC for the test bacteria [50].

The existence of viable bacteria colonies after 24 h of incubation with the antibacterial agent present could be due to the occurrence of mutant forms which resist and grow in the presence of extract concentrations and change to vegetative forms when antibacterial agents are withdrawn. Mutant bacterial cells devoid of cell wall can be produced by both Gram-negative and Gram-positive bacteria [14]. This could also be due to resistance as a result of biofilm formation, since microbial populations adhered to surfaces are physiologically different from planktonic cells of the same bacteria [13].

## 5. Conclusions and Recommendations

The dichloromethane: methanolic extract of *C. asiatica* showed antimicrobial activities. These findings imply that *C. asiatica* may be a potential candidate to obtain an antimicrobial agent in the management of infectious diseases. This study, therefore, confirms the antibacterial activities of *C. asiatica* and supports its use against bacterial infections in the Gusii community in Kenya and other parts of the world.

Based on the abovementioned conclusion, the following recommendations are forwarded:

- The plant part should be extracted with other solvent types to get enough of bioactive molecules
- The different fractions of the plant extract to be evaluated for antibacterial activities

## Figures and Tables

**Table 1:** Zones of inhibition produced by the *C. asiatica* extract against bacteria strains in mm.

Group	Zones of inhibition (mm)					
	Treatment	*Staphylococcus aureus*	*Escherichia coli*	*Salmonella typhi*	*Bacillus subtlis*	*Shigella sonnei*
Negative control	DMSO	6.00 ± 0.00^d^	6.00 ± 0.00^f^	6.00 ± 0.00^e^	6.00 ± 0.00^d^	6.00 ± 0.00^d^

Positive control	Tetracycline	29.33 ± 0.67^a^	26.67 ± 0.33^a^	27.67 ± 0.33^a^	22.67 ± 0.33^a^	25.33 ± 0.33^a^

*C. asiatica* DCM: MeOH extract	15.625 mg/ml	6.00 ± 0.00^d^	6.00 ± 0.00^f^	6.00 ± 0.00^e^	6.00 ± 0.00^d^	6.00 ± 0.00^d^
	31.25 mg/ml	6.00 ± 0.00^d^	6.00 ± 0.00^f^	6.00 ± 0.00^e^	6.00 ± 0.00^d^	6.00 ± 0.00^d^
	62.5 mg/ml	6.00 ± 0.00^d^	7.33 ± 0.33^e^	6.00 ± 0.00^e^	6.00 ± 0.00^d^	6.00 ± 0.00^d^
	125 mg/ml	6.00 ± 0.00^d^	8.67 ± 0.33^d^	8.00 ± 0.33^d^	6.00 ± 0.00^d^	7.33 ± 0.33^d^
	250 mg/ml	9.67 ± 0.33^c^	13.67 ± 0.33^c^	10.67 ± 0.33^c^	7.67 ± 0.33^c^	9.00 ± 0.58^c^
	500 mg/ml	12.00 ± 0.58^b^	16.33 ± 0.33^b^	13.00 ± 0.58^b^	9.67 ± 0.33^b^	15.67 ± 0.33^b^

Values are expressed as mean ± standard error of the mean (SEM) for triplicate reading. Values with the same superscript letter in the columns are not significantly different by one-way ANOVA followed by Tukey's post-hoc test.

**Table 2:** Minimum inhibitory concentrations (MICs) and minimum bactericidal concentrations (MBCs) for bacteria test cultures in mg/ml.

	Concentration			
	*C. asiatica* (mg/ml)		Tetracycline (*µ*g/ml)	
	MIC	MBC	MIC	MBC
*S. typhi*	62.50 ± 0.00^a^	125.00 ± 0.00^a^	1.96 ± 0.22^b^	3.91 ± 0.00^b^
*S. sonnei*	52.1 ± 10.40^ab^	62.50 ± 0.00^b^	4 ± 0.00^a^	32 ± 0.00^a^
*B.subtilis*	52.1 ± 10.40^ab^	62.50 ± 0.00^b^	1.96 ± 0.22^b^	15.63 ± 0.1^a^
*E.coli*	62.50 ± 0.00^a^	125.00 ± 0.00^a^	0.98 ± 1.0^c^	3.91 ± 0.00^b^
*S.aureus*	26.04 ± 5.21^b^	52.10 ± 10.4^b^	0.25 ± 0.00^c^	1.96 ± 0.00^c^

Values are expressed as mean ± standard error of the mean (SEM) for triplicate reading. Values with the same superscript letter in the columns are not significantly different by one-way ANOVA followed by Tukey's post-hoc test.

**Table 3:** Phytochemical composition of the dichloromethane:methanolic extract of *C. asiatica*.

Phytochemical	*Centella asiatica*
Alkaloids	+
Flavonoids	+
Steroids	−
Saponins	+
Cardiac glycosides	+
Phenolics	+
Terpenoids	+
Tannins	+

Present phytochemicals are denoted by the (+) sign, while the absent phytochemicals are denoted by the (−) sign.

## Data Availability

The data used to support the findings of this study are available from the corresponding author upon request.

## Appendix

### Procedures for the Preparation of Standards

#### Procedures to Prepare 0.5 McFarland Standard Solutions

Original *McFarland* standards were made by mixing specified amounts of barium chloride and sulfuric acid together. Mixing the two compounds forms a barium sulfate precipitate, which causes turbidity in the solution. A 0.5 McFarland standard is prepared by mixing 0.05 ml of 1.175% barium chloride dihydrate (BaCl_2_∙2H_2_O) with 9.95 ml of 1% sulfuric acid (H_2_SO_4_). The standard can be compared visually to a suspension of bacteria in sterile saline or nutrient broth. If the bacterial suspension is too turbid, it can be diluted with more diluents. If the suspension is not turbid enough, more bacteria can be added.
